# Deep Learning-Based Multiclass Instance Segmentation for Dental Lesion Detection

**DOI:** 10.3390/healthcare11030347

**Published:** 2023-01-25

**Authors:** Anum Fatima, Imran Shafi, Hammad Afzal, Khawar Mahmood, Isabel de la Torre Díez, Vivian Lipari, Julien Brito Ballester, Imran Ashraf

**Affiliations:** 1 National Centre for Robotics, National University of Sciences and Technology (NUST), Islamabad 44000, Pakistan; 2 College of Electrical and Mechanical Engineering, National University of Sciences and Technology (NUST), Islamabad 44000, Pakistan; 3 Military College of Signals (MCS), National University of Sciences and Technology (NUST), Rawalpindi 44000, Pakistan; 4 Department of Signal Theory and Communications and Telematic Engineering, University of Valladolid, Paseo de Belén 15, 47011 Valladolid, Spain; 5 Research Group on Foods, Nutritional Biochemistry and Health, Universidad Europea del Atlántico, Isabel Torres 21, 39011 Santander, Spain; 6 Department of Project Management, Universidad Internacional Iberoamericana, Campeche 24560, Mexico; 7 Fundación Universitaria Internacional de Colombia Bogotá, Bogotá 11001, Colombia; 8 Department of Project Management, Universidad Internacional Iberoamericana Arecibo, Arecibo, PR 00613, USA; 9 Project Management, Universidade Internacional do Cuanza, Cuito EN250, Bié, Angola; 10 Department of Information and Communication Engineering, Yeungnam University, Gyeongsan 38541, Republic of Korea

**Keywords:** Mask-RCNN, MobileNet, deep learning, dental disease detection

## Abstract

Automated dental imaging interpretation is one of the most prolific areas of research using artificial intelligence. X-ray imaging systems have enabled dental clinicians to identify dental diseases. However, the manual process of dental disease assessment is tedious and error-prone when diagnosed by inexperienced dentists. Thus, researchers have employed different advanced computer vision techniques, as well as machine and deep learning models for dental disease diagnoses using X-ray imagery. In this regard, a lightweight Mask-RCNN model is proposed for periapical disease detection. The proposed model is constructed in two parts: a lightweight modified MobileNet-v2 backbone and region-based network (RPN) are proposed for periapical disease localization on a small dataset. To measure the effectiveness of the proposed model, the lightweight Mask-RCNN is evaluated on a custom annotated dataset comprising images of five different types of periapical lesions. The results reveal that the model can detect and localize periapical lesions with an overall accuracy of 94%, a mean average precision of 85%, and a mean insection over a union of 71.0%. The proposed model improves the detection, classification, and localization accuracy significantly using a smaller number of images compared to existing methods and outperforms state-of-the-art approaches.

## 1. Introduction

Over the past decade, artificial intelligence (AI) has made remarkable contributions to various subdisciplines falling under the category of dentistry, specifically periodontology. Different studies have explored dental disease detection, localization, classification, and segmentation within the dental domain (e.g., [1]). However, few studies have explored dental disease localization as discussed in the literature. From the existing literature, several challenges are found regarding dental carious region localization. A comprehensive overview of existing studies is presented in Table 1. Further exploration is required to propose detection and localization approaches for dental caries diagnosis in real time.

To classify enamel, dentin, and pulp caries, Oprea et al., proposed rule-based classification. The authors were able to categorize regions as dentin caries sized over 2 mm [9]. Another rule-based approach based on the gradient histogram and threshold was proposed by ALbahbah and fellow authors on panoramic radiographs to extract and segment decayed and normal teeth [10]. Lin et al., investigated the level segmentation method based using SVM (support vector machine), KNN (K nearest neighbor), and a Bayesian classifier for localizing alveolar bone loss [11]. Results show that the model can localize alveolar bone loss with higher classification accuracy. A cluster-based segmentation technique was proposed by Datta and Chaki to detect dental cavities in [12]. The proposed model utilized a Wiener filter to extract caries lesions followed by region segmentation to monitor the lesion size and growth. To detect and classify proximal carious and non-carious lesions on panoramic radiographs, Na’am et al., explored multiple morphological gradient-based image processing methods on images with manually cropped regions [13].

Different deep learning approaches have been employed by researchers to pave way for more efficient and effective methods to diagnose dental caries. To classify carious and non-carious teeth on a small labeled dataset, a pre-trained CNN was utilized by Prajapati et al. [14]. The model was able to classify dental caries, periodontitis, and periapical infection. Lee et al. utilized a deep CNN to diagnose and classify caries using 3000 periapical radiographs [15]. The model achieved an AUC of 0.91 for premolar, 0.89 for molar, and 0.84 for both premolar and molar models. For the identification of dental caries, Cantu et al., investigated U-Net on bitewing radiographs [16]. It was found that segmentation-based models possess the potential to aid dental clinicians in detecting and locating dental caries more efficiently. For the identification of endo-perio lesions on periapical radiographs, Sajjad et al., investigated AlexNet, for which the model achieved an accuracy of 98% [17]. For early identification of dental caries, Kumari et al., preprocessed bitewing radiographic images using contrast limited adaptive histogram equalization (CLAHE) and noise filtering followed by a meta-heuristic based ResneXt RNN (recurrent neural network) [18].

Radiological examinations help dental clinicians in the identification of teeth abnormalities, cysts, infections, and infections. However, manual examinations are time-consuming and rely solely on a specialist’s opinion which may bring differences in the diagnosis. Different methods have been employed by researchers in recent years mainly relying on boundary-based, region-based [19], cluster-based, and threshold-based methods [11]. As the first step, Jader et al., employed an RCNN for the segmentation of caries and the detection of missing teeth on buccal images. The results indicated that deep learning-based instance segmentation has the potential to automate the process of caries detection and medical report generation [2].

The faster region-based convolutional neural network (Faster-RCNN), which extends the Fast-RCNN is utilized to localize teeth lesions [5]. The model achieves both a recall and precision of above 90%, however, the model suffers in numbering the teeth in complicated cases. A Faster-RCNN built on the region proposal network (RPN) and object detection network (ODN) detected different types of teeth achieving a mean average precision (mAP) of 91.40% and an accuracy of 91.03% [6]. However, the model was applied to a small dataset and performance can not be generalized. Another variant of Faster-RCNN pre-trained on ResNet-50 was employed in [7] for the detection of carious teeth, achieving a precision of 73.49% and an F1 score of 0.68. The model, however, does not identify the type of caries and only localizes the caries region.

An M-RCNN, which extends the Faster-RCNN with pre-trained ResNet-101 was found to be helpful in the identification of missing or broken teeth, achieving an accuracy of 98% [2]. However, segmentation performance metrics were not reported in the study. For pixel-wise segmentation of visible light images for identification of oral cavities [3], M-RCNN achieves an accuracy of 74.4%. However, the dataset is sparse and other relevant performance metrics have not been reported for comparison. In another attempt, an M-RCNN with a fully convolutional network (FCN) and a ResNet-101 backbone [4] was investigated to localize occlusal surface caries on a limited dataset, but the computational complexity was not reported. In a recent attempt, a hybrid M-RCNN [8] was employed to identify dental caries on mixed images achieving an average precision of 81.02% and an accuracy of 95.75%, however, the model does not identify caries type for both colored and X-ray images. Additionally, an M-RCNN with ResNet as its backbone requires a substantial amount of calculations to learn and analyze, and the training process for M-RCNN requires high-performance computational resources such as GPU and memory [20].

There are very few studies focusing on usable carious region detection and localization on periapical radiographs. The existing approaches for dental lesion localization provide the key knowledge, which can be adopted by researchers to focus on implementing improved segmentation and localization approaches for dental caries. This research aims to

- Put forward an automated deep learning-based dental caries localization and segmentation model to identify the type of periapical lesion and localize the lesion on periapical radiographs,
- Propose a lightweight MobileNet-v2 with additional layers to enhance the performance of the Mask-RCNN on a small periapical dataset,
- Preprocess low-resolution images to obtain better disease diagnosis performance,
- Provide a comprehensive evaluation and comparison of state-of-the-art deep learning-based segmentation and localization methods with the proposed model,
- Introduce an annotated dental lesion dataset to identify periapical lesions, considering the limitation of data availability.

The remainder of the paper is organized into four sections. The proposed methodology is described in Section 2. Section 3 discusses the experimental results, while the conclusion is provided at the end.

## 2. Proposed Methodology for Periapical Disease Detection, Classification, and Localization

**Figure 1:**
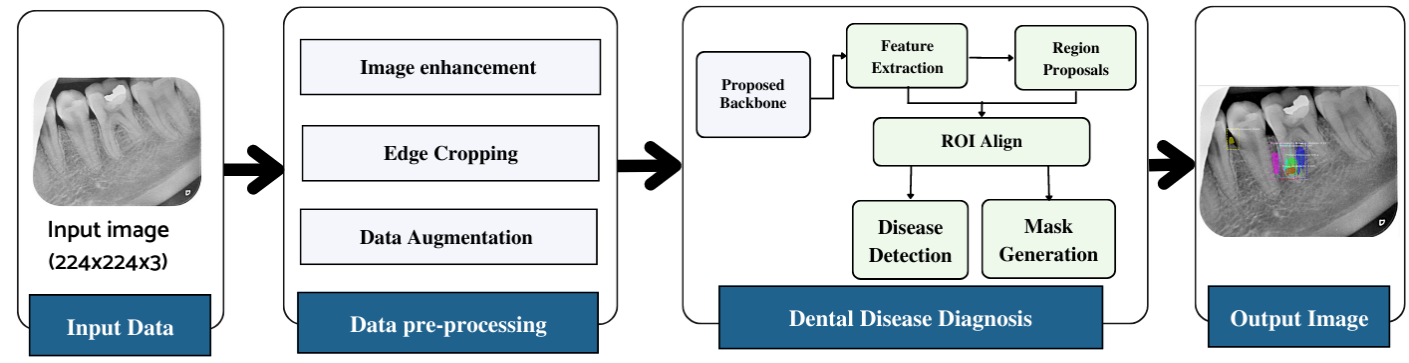
Workflow diagram of the proposed approach.

The workflow of the proposed dental lesion detection process is shown in Figure 1. First, the collected annotated images are preprocessed to remove noise, enhance contrast, and improve the resolution of the images. Next, the preprocessed images are used by the proposed lightweight backbone network for feature extraction. The extracted feature maps are then forwarded to the RPN that generates region proposals using the feature maps and forwards them to the region of interest (ROI) align block. This block processes both the feature maps and region proposals and classifies the input image using fully connected layers. The model further exhibits the bounding box on the identified region so it can be visualized.

### 2.1. Dataset Analysis and Preprocessing

**Figure 2:**
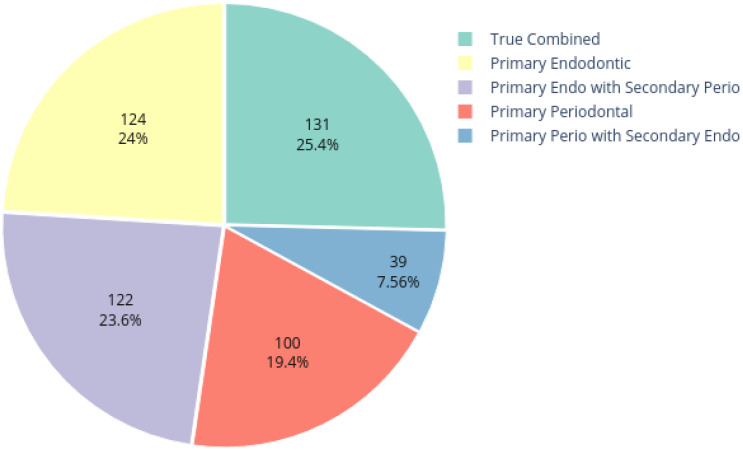
Dataset distribution.

The dataset employed for this study was obtained from the Armed Forces Institute of Dentistry, Rawalpindi Pakistan. A total of 534 periapical images were collected, out of which 516 were labeled by experienced radiologists and dentists. The dataset distribution is shown in Figure 2. The dataset includes radiographs with only periapical lesions.

Inclusion criteria were teeth surrounded by alveolar bone in either the upper or lower jaws and radiographs with signs of periapical lesions. The exclusion criteria comprised teeth with radiographic evidence of any other lesion, radiographs that included both upper and lower jaws, radiographs rated as unacceptable due to positioning and processing, and issues with exposure and visibility.

**Figure 3:**
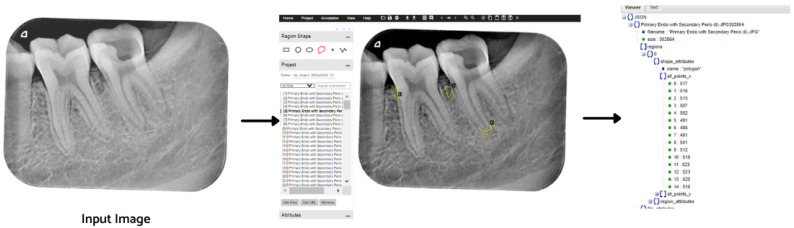
Image labeling process using the VIA tool.

The ground truth of the obtained images was generated using a VGG Image Annotator (VIA) tool [21]. Five types of lesions were localized manually using bounding polygons around the carious regions. The annotations were saved in a JSON file, where each mask represents a set of polygon points. The pixels inside the bounding polygons corresponding to lesions were assigned values of 1 for primary endodontic, 2 for primary endo with secondary perio, 3 for primary periodontal, 4 for primary perio with secondary endo, and 5 for true combined, while the rest of the pixels were regarded as background with a value of 0. For each labeled data point, there is corresponding instance information as illustrated in Figure 3.

The annotated image dataset is preprocessed to improve image quality and remove noise in the radiographic images. The influence of image preprocessing has been analyzed in several studies. Tian et al., found that image enhancement leads to better performance of Fast-RCNN for detection tasks [22]. Chen et al., evaluated image enhancement on RGB images followed by a deep learning-based method for accurate prediction of retinal blood vessels [23]. In a more recent attempt, Pannetta et al. [24] tested different image enhancement techniques such as histogram equalization (HE) [25], contrast limited adaptive histogram equalization (CLAHE) [26], dynamic fuzzy histogram equalization (DFHE) [27], guided filtering (GF) [28], and bi-histogram equalization (BBHE) [29] on a medical image dataset. It was found that CLAHE performs better in comparison to other techniques to enhance the contrast of the images. This study utilizes CLAHE to enhance image contrast to improve the performance of the proposed lightweight disease detection and localization model.

### 2.2. Lightweight Mask RCNN

An M-RCNN requires higher computing resources for training in order to learn and analyze substantial information obtained from medical imagery. To reduce the computational requirements of M-RCNN and ensure that it operates properly, a lightweight backbone network is utilized with the M-RCNN to classify five types of endo-perio lesions. The focus of this research is to propose a lightweight M-RCNN model that can operate on platforms with less computational resources such as graphics processing unit (GPU) and memory and provide performance similar to that of the original M-RCNN [20].

**Figure 4:**
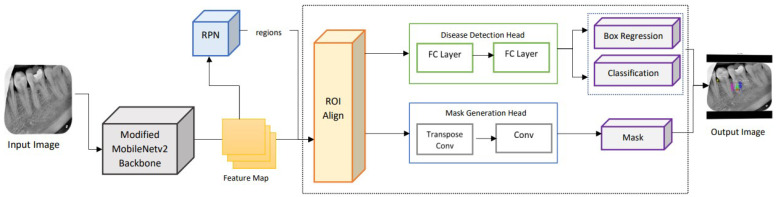
Architecture of the lightweight M-RCNN modified with MobileNet-v2 as its backbone.

For this purpose, a lightweight network MobileNet-v3 is utilized for feature extraction followed by a depthwise separable convolutional layer proceeding a tiny RPN to extract candidate regions with potential targets [30]. The RPN generates anchor boxes for each classified object using the softmax activation function. The extracted proposal regions, along with feature maps, are applied to ROI alignment to locate all the feature map areas. ROI alignment wraps different feature vectors, which are then applied to mask generation and classification. The fully connected layer provides classification and bounding boxes for each identified endo-perio lesion. The masks are generated by the convolution layer for each object at the pixel level. The proposed framework for the lightweight M-RCNN for dental lesion classification and localization is depicted in Figure 4.

#### 2.2.1. Backbone Network

**Figure 5:**
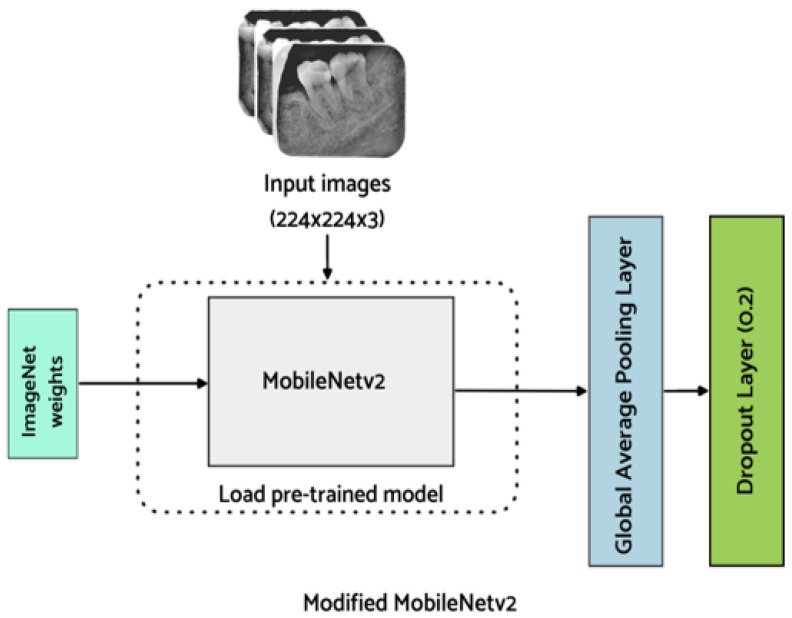
Proposed backbone network (Modified MobileNet-v2).

To reduce the number of parameters in the proposed lightweight M-RCNN, MobileNet-v2 is employed, which extends MobileNet-v1 and is faster with 30% fewer parameters [31]. In MobileNet-v2 [32], an inverted residual structure is introduced to reduce complexity and increase the speed. The employed backbone network comprises two layers with the first layer of 1 × 1 pointwise convolution with ReLu6 and a depthwise convolutional layer. The inverted design of the employed MobileNet-v2 makes the model considerably more memory efficient and improves overall performance. The structure of the employed MobileNet-v2 is illustrated in Figure 5.

For the classification of endo-perio lesions, MobileNet-v2 modified with additional layers, as proposed by Kolonne et al. [33], is utilized in this work. To avoid impairment of already learned features, the base layers are frozen. Additionally, the fully connected layer of MobileNet-v2 is replaced with a global average pooling layer which averages the feature map in the convolutional layers. Additionally, a dropout layer is added to minimize the model from overfitting. Finally, a dense layer is added for the classification of endo-perio lesions. The model weights are saved after fine-tuning the hyperparameters of the model to improve the classification results. As this is a multi-class classification problem, softmax is used as an activation function in the output layer to predict the probability for each of the five classes and is defined below

(1) $$\sigma \left(z\right)=\frac{1}{1+e^{-z}}$$

#### 2.2.2. Region Proposal Network

**Figure 6:**
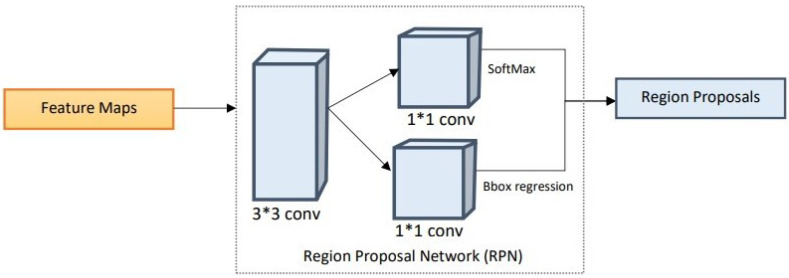
Structural diagram of the region proposal network.

Once the multi-scale features are extracted using the proposed lightweight backbone network, the feature maps are passed onto the RPN. The RPN performs matching of detected regions to the ground truth. The region proposals are predicted simultaneously in each sliding window, where *k* represents the maximum possible proposals at each location. Additionally, *k* proposals are parameterized for each proposal to form anchors [34]. Due to the small size of the regions to be localized in the periapical radiographs, anchor sizes and anchor aspect ratios are based on extensive experimentation to adequately fit the task at hand [35]. The anchors are matched to the ground truth regarding the intersection over union (IoU) between the anchor and the ground truth. The anchors are linked to the ground truth boxes and are assigned to the foreground once the IoU exceeds the defined threshold, which is 0.7 in this study. If the IoU is below the defined threshold, the identified region is ignored. The proposal regions with an IoU higher than the threshold are considered as foreground. This block provides several ROIs that are then utilized by ROI alignment to identify where these regions of interest lie in the feature maps. The structure diagram of the RPN is illustrated below in Figure 6.

#### 2.2.3. Region of Interest Alignment

The ROI align block extracts feature vectors from the feature map based on the regions of interest identified by the RPN [36]. These feature vectors are turned into fixed-sized tensors to be processed further. The ROI is scaled with their corresponding areas based on the regions’ location, scales, and aspect ratios. To ensure uniformity, the samples are aligned over feature map areas. After generating the region proposals, the next block involves making predictions by taking ground truth boxes, feature maps generated by the proposed lightweight backbone network, and region proposals generated by the RPN. Additionally, the results represented by ROI feature maps are then processed by two parallel branches: disease detection and mask generation.

- Disease Detection Head: Using the ROI feature map, the disease category is predicted along with the refined instance boundary box. This branch contains two fully connected (FC) layers to map the feature vector to the classes and instance bounding box coordinates.
- Mask Generation Head: The ROI feature map is fed into a transposed convolutional layer followed by another convolutional layer. The segmentation masks are generated for the classes and the output mask is selected according to the class prediction provided by the disease detection branch.

### 2.3. Loss Function

A multiclass loss function for the proposed lightweight Mask-RCNN is used, which combines the loss of classification, localization, and segmentation mask and is calculated as shown in Equation (2)

(2) $$L=L_{cls}+L_{box}+L_{mask}$$

where $L_{cls}$ and $L_{box}$ are similar to the Faster-RCNN and are used as loss functions in both BBox regression and classification. Additionally, $L_{mask}$ generates a mask with dimensions K×m×m for each ROI extracted after the RPN and classifies each pixel for corresponding classes and *K* represents the number of classes to be classified, which is five in this case.

### 2.4. Performance Measure

The performance of the proposed model is measured based on different performance indicators. For the evaluation of the model’s classification, classification accuracy, sensitivity, and specificity are chosen. The area under the receiving operating characteristic curve (AUC). To detect and evaluate the detection performance of the proposed model, mean average precision (mAP), mean average recall (mAR), and F1 score are chosen. Average precision (AP) is calculated for each class and then the average is taken over N classes to calculate mAP [37]. A trade-off between precision and recall is considered along with both false positive (FP) and false negative (FN) results. The calculation of mAR is similar to mAP, however, the recall for mAR is calculated for different IoU thresholds [38] and is calculated as two times the area under the recall IoU curve averaged over 241 IoUs, ranging between 0.5 and 1. After calculating mAP and mAR, the F1-score is calculated using mAP and mAR, respectively.

## 3. Experiments and Results

For comparison, the proposed Mask-RCNN is based on a lightweight MobileNet-v2, and the base RCNN is trained under the same environment. In the collected dataset, each image belongs to one of the disease classes. The custom dataset is used to train the proposed model. The images within the dataset are divided as 80% for training, 10% for validation, and the remaining 10% for testing. The images within the dataset belong to one of the disease types

- Primary endo with secondary perio,
- Primary periodontal,
- Primary perio with secondary endo,
- Primary endodontic, and
- True combined

To reduce the computational time and increase the efficiency of Mask-RCNN, a modified lightweight pre-trained Mobilenet-v2 is employed as the backbone network of Mask-RCNN. Different values for hyperparameters are employed to see the performance of the proposed model on the disease detection dataset. To ensure an effective comparative experiment, the hyperparameters for testing different backbone networks with Mask-RCNN are kept consistent. The following subsections explain the experimental settings, the ablation experiment conducted to see how the proposed model performs with different backbone networks, and the performance evaluation of the proposed model on the test dataset.

### 3.1. Experiment Setting

For conducting experiments, a laptop equipped with an Intel i7-1165G7 processor (2.80 GHz), and 8GB RAM is utilized. Additionally, the code was implemented on Google Colab equipped with Python 3.5, Tensorflow 1.14.0, and Keras 2.2.5. The configurations employed in the implementation of the model are shown in Table 2.

### 3.2. Dataset Preprocessing

**Figure 7:**
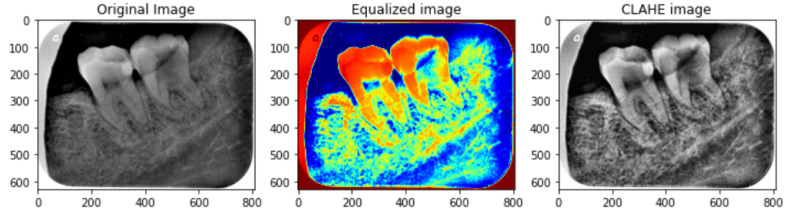
Preprocessed image sample using CLAHE. The left image is the original image, the middle image is color equalized image while the right image is the final CLAHE image.

To enhance the small details, local contrast, and texture of medical images, CLAHE [39] is used. Different tile regions of the image based on the histogram are computed using CLAHE. The local details of the radiograph are enhanced by limiting histogram amplification and clipping of the histogram. Additionally, CLAHE allows reducing over-amplification of noise within X-ray images and serves as a better alternative for image enhancement compared to manual delineation methods [40]. The process of CLAHE is carried out in two steps. First, the image is divided into non-overlapping regions that are equal in size followed by obtaining the clip limit for the clipping histogram. In the second step, the histogram is redistributed so that the height remains under the clip limit. The results obtained using CLAHE are illustrated in Figure 7.

**Figure 8:**
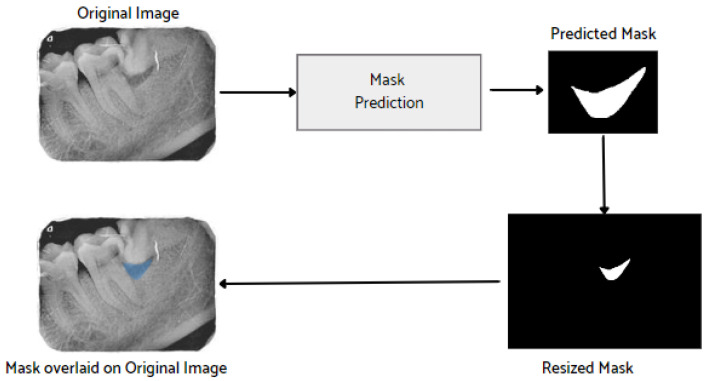
Mask overlaid on the original image.

After the image has been preprocessed, the mask generated for the images from the JSON file in the collected dataset is overlaid on the original images. A visualization of the mask predicted using the proposed M-RCNN on the collected disease dataset is illustrated in Figure 8.

### 3.3. Experiments

**Figure 9:**
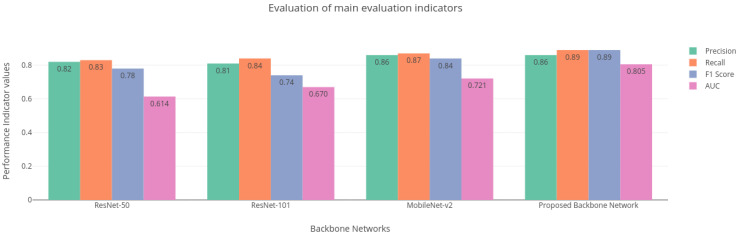
Main evaluation indicators for different backbone networks.

To further examine the effectiveness and contribution of the proposed method, additional ablation experiments are conducted [41]. The aim of the ablation experiment is to provide deeper insights into the improvements obtained by the proposed model. The proposed model is built and trained using Tensorflow by Google US which is an open-resource deep learning application programming interface (API). For comparison of the backbone networks, the hyperparameters are kept consistent (optimizer ’Adam’ is chosen with a learning rate of 0.0001 and loss function ’categorical cross entropy’). Additionally, to prevent the model from overfitting, early stopping is applied. The results obtained from the ablation studies are shown in Table 3. Additionally, the comparison of the experimental results using different backbones is shown in Figure 9.

The performance of the base Mask-RCNN is evaluated with regard to the evaluation metrics such as precision, recall, mAP, and AUC. It is evident from Table 3 that the proposed modified lightweight Mobilenet-v2 performs accurately compared to other models like ResNet-50 and ResNet-101 that are employed by the base Mask-RCNN, achieving an overall precision of 0.86, recall of 0.89, mAP of 0.85, and ROC AUC of 0.805 for detection and classification of five types of disease while reducing the number of parameters compared to the base Mask-RCNN.

Additionally, the performance of the proposed model was evaluated based on well-known performance indicators. The result of the proposed model for each classified disease is shown in Table 4. The performance parameters indicate that the proposed backbone network provides good performance for disease classification.

### 3.4. Performance Evaluation

Different studies have employed an RCNN to segment and detect caries [2,3,4,7,18]. However, these methods provide less precision for detecting very small lesions within a periapical radiograph. The proposed lightweight periapical lesion detection system is able to detect very small periapical lesions (a difficult task for presently available AI-based systems) with increased precision and is able to distinguish teeth with small lesions from teeth without any lesions in a periapical radiograph. To verify the effectiveness of the model, two performance indicators mAP and IoU are used. The results from Table 5 indicate that the proposed Mask-RCNN with the lightweight modified MobileNet-v2 backbone network and RPN-based region detection achieved an mAP of 0.85, which is higher compared to other models. Mask-RCNN with ResNet-50 [18] achieves an mAP of 0.71, ResNet-101 [2,3] achieves an mAP of 0.74, ResNet-101 with FCN-based region detection [4] has an mAP of 0.67 and Faster-RCNN with ResNet-50 [7] obtains an mAP of 0.80. Results suggest that the proposed approach outperforms other models.

#### Comparison with Different Backbone Networks

To demonstrate the performance superiority of the proposed model, the previous section discusses the experiments conducted to show the performance comparison of different backbone networks with an M-RCNN based on consistent hyperparameters. The results indicate that the proposed backbone network performs better than other state-of-the-art networks employed as the backbone with M-RCNN. However, to further validate the proposed model’s precision and accuracy, it is compared with the base M-RCNN, ResNet-50, and ResNet-101 backbone networks. The proposed and comparison model is tested on different periapical radiographic images.

For the evaluation of the proposed lightweight M-RCNN, the collected dataset is divided into train, valid, and test sets. In the dataset, for comparison, the proposed model is compared with the base M-RCNN using the ResNet-50 as a backbone network and M-RCNN using the ResNet-101 as a backbone network. The parameter configuration for the experiment is shown in Table 2. For training, the learning rate was set to 0.01, which was later adjusted to 0.001, the weight decay was set to 0.0001, while the learning momentum was 0.9. In medical images, the localized regions are often smaller in size, so to fit the disease regions more accurately in this study, the RPN anchor scale was set to (8, 16, 64, 128, 256), and the BBOX standard deviation to [0.1 0.1 0.2 0.2]. The model is trained for 50 epochs, and due to the small dataset, the batch size was kept at 2 with 50 validation steps, as well as 200 steps per epoch.

### 3.5. Comparison with Test Images

**Figure 10:**
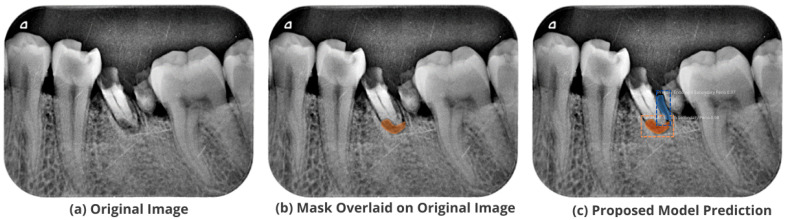
Test performance for the localization of a ’Primary Endo and Secondary Perio’ lesion.

**Figure 11:**
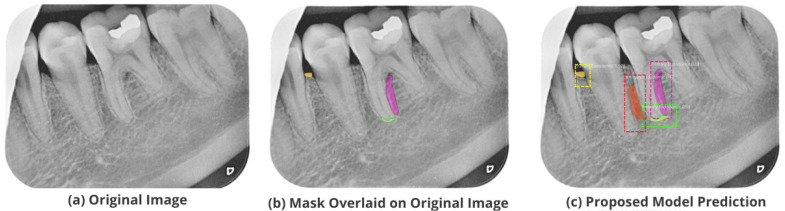
Test performance for the localization of a ’Primary Endodontic’ lesion.

**Figure 12:**
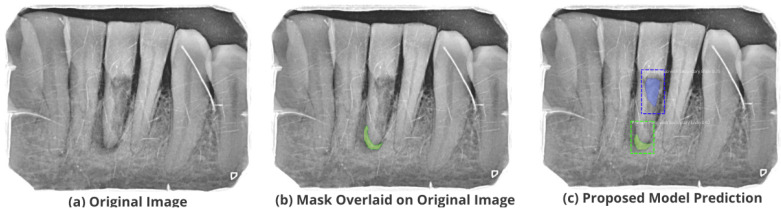
Test performance for the localization of a ’Primary Perio and Secondary Endo’ lesion.

**Figure 13:**
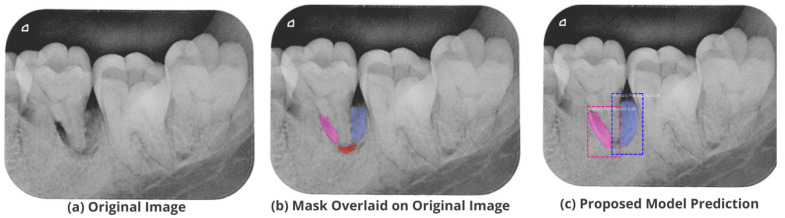
Test performance for the localization of a ’Primary Periodontal’ lesion.

**Figure 14:**
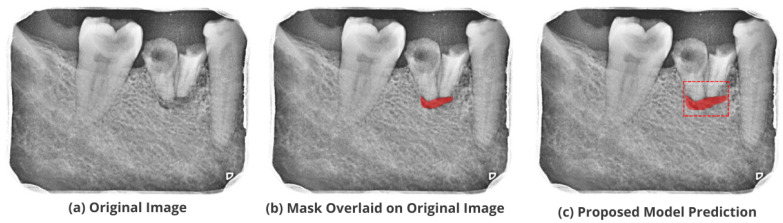
Test performance for the localization of ’True Combined’ lesions.

The proposed model accurately predicts and localizes the lesions as depicted in Figure 10, Figure 11, Figure 12, Figure 13 and Figure 14. The results indicate that the model makes predictions similar to that of the annotated masks using the periapical radiographic images. Additionally, the proposed model was evaluated based on performance indicators like precision, recall, F1 score, and accuracy for each classified periodontal lesion. The obtained results indicate that the proposed backbone network provides good performance for disease classification. All the test images for the proposed study show a 95% confidence level indicating that the database annotations are in the right direction.

Authors: high definition figures are not available.

## 4. Conclusions and Future Directions

This study proposes a detection and localization network based on deep learning for the classification and localization of different periodontal lesions in periapical radiographic images. For feature map extraction, a lightweight modified MobileNet-v2 is utilized by adding a global average pooling layer, as well as a dropout layer to enhance the performance of the Mask-RCNN model followed by a region proposal network for the identification of region proposals. The proposed mechanism provides multi-disease prediction by obtaining anchor boxes. Additionally, hyperparameters are fine-tuned to further improve the performance of the model and acquire accurate predictions. The presented system detects periapical lesions that are tough to recognize by other existing methods due to the complex nature of radiographic images. The images are preprocessed using CLAHE to enhance image contrast and reduce noise to gain better performance. The proposed model is tested on a custom-made dataset with annotated disease labels. The masks are generated using the provided annotations which are then utilized to train the model. The results indicate that the proposed model is capable of enhanced identification and localization of periapical disease with increased precision superior to other existing dental disease localization solutions in radiographic images.

There are certain limitations to the proposed approach that need further research to improve its performance. The dataset used to train the proposed model is small in terms of size. In this work, image augmentation is used for better classification accuracy. Other techniques such as generative adversarial networks can be employed to generate synthetic data. Additionally, the model’s performance may improve further by employing a larger annotated dataset with multiple lesions. This work can be extended further by embedding the Internet of things (IoT) for data collection and making the proposed mechanism widely accessible. The proposed model’s performance can be analyzed on other radiographic datasets such as panoramic radiographs, colored images, and a hybrid dataset combining both radiographic and colored images.

## Figures and Tables

**Table 1:** Strengths and weaknesses of baseline dental lesion localization models.

Study	Image Modality	Task	Method	Strengths	Weaknesses
Jader et al., 2018 [2]	Panoramic images	Localize missing teeth	M-RCNN with ResNet-101 backbone	The model is helpful in identifying missing or broken teeth with an accuracy of 98%	- Highly variable data - Other metrics i.e., mAP, IoU are not reported for comparison
Anantharaman et al., 2018 [3]	Colored images	Detect and segment cold/canker sores	M-RCNN with ResNet-101 backbone	The model is helpful in performing pixel- wise segmentation of visible light images of oral cavity with accuracy of 74.4%	- Sparse dataset - Other metrics i.e, mAP, IoU, precision, F1-score, recall are not reported for comparison
Moutselos et al., 2019 [4]	Colored images	Localize and classify caries occlusal surfaces	M-RCNN with FCN and ResNet-101 backbone	The model provided encouraging performance for automatically selecting image texture features and detect lesions without additional pre-processing actions	- The computational complexity is not reported
Chen et al., 2019 [5]	Periapical radiographs	Teeth localization and numbering	Faster-RCNN	The model detects and numbers teeth with recall and precision exceeding 90% on manually annotated dataset	- The model suffers in numbering teeth in complicated cases such as heavily decayed teeth
Laishram & Thongam, 2020 [6]	Panoramic radiographs	Localize and classify different type of teeth	Faster-RCNN built on RPN and ODN	The model is helpful in detecting different types of teeth achieving mean average precision (mAP) of 91.40% and accuracy of 91.03%	- Limited dataset in terms of size
Zhu et al., 2022 [7]	Periapical radiographs	Detection of carious teeth	Faster-RCNN with pretrained ResNet-50	The model is helpful in with an average precision of 73.49%, F1-score of 0.68 with sample detection speed of 0.1923	- It suffers from computational compexity - The model does not identify caries type
Rashid et al., 2022 [8]	Mixed images (colored and periapical radiographic images)	Detect and localize dental carious regions	Hybrid M-RCNN	The model was helpful in localizing dental carious regions with a precision of 81.02% and accuracy of 95.75%	- Limited dataset in terms of size - The model does not identify caries type for both colored and X-ray image

**Table 2:** Parameter configuration for this experiment.

Weight Decay	Learning Rate	Min Detect Confidence	Epoch	Batch Size
0.0001	0.001	0.7	50	2
**Validation Steps**	**Steps per Epoch**	**Learning Momentum**	**RPN Anchor Scale**	**BBOX Standard Deviation**
50	200	0.9	(8, 16, 64, 128, 256)	[0.1 0.1 0.2 0.2]

**Table 3:** Comparison of M-RCNN with different backbone networks.

Model	Backbone Network	Precision	Recall	F1-Score	ROC AUC
Mask-RCNN	ResNet-50 [19]	0.82	0.83	0.78	0.614
	ResNet-101 [42]	0.81	0.84	0.74	0.670
	MobileNet-v2 [43]	0.86	0.87	0.84	0.721
	Proposed Backbone Network	0.86	0.89	0.89	0.805

**Table 4:** Comparison of performance indicators for each disease.

Class	Accuracy	Precision	Recall	F1-Score
Primary Endo with Secondary Perio	0.89	0.83	0.75	0.77
Primary Periodontal	0.96	0.80	1.00	0.75
Primary Perio with Secondary Endo	0.87	0.91	0.90	0.91
Primary Endodontic	0.92	0.94	0.86	0.89
True Combined	0.97	0.87	0.89	0.88
Average	0.93	0.86	0.89	0.89

**Table 5:** Comparison of the measurement index of different networks for disease localization.

Model	Variation	Mean Average Precision (mAP)	Mean Insection over Union (mIoU)
Mask-RCNN	ResNet-50 [19]	0.71	0.70
	ResNet-101 [2,3]	0.74	0.68
	ResNet101 backbone and FCN-based region detection [4]	0.67	0.58
Faster RCNN	ReNet-50 [7]	0.80	0.69
Mask-RCNN	Lightweight Modified MobileNet-v2 backbone with RPN-based region detection	0.85	0.71

## Data Availability

The dataset and code is available from the corresponding author on reasonable request.
